# Inhibitory Effect of Waste Glass Powder on ASR Expansion Induced by Waste Glass Aggregate

**DOI:** 10.3390/ma8105344

**Published:** 2015-10-09

**Authors:** Shuhua Liu, Shu Wang, Wan Tang, Ningning Hu, Jianpeng Wei

**Affiliations:** 1 State Key Laboratory of Water Resources and Hydropower Engineering Science, Wuhan University, Wuhan 430072, China; wangshu@whu.edu.cn (S.W.); tangwan@whu.edu.cn (W.T.); huningning@whu.edu.cn (N.H.); weijianpeng@whu.edu.cn (J.W.); 2 Department of Civil, Environmental and Geomatic Engineering, University College London, Gower Street, London WC1E 6BT, UK; 3 China Construction Ready Mixed Concrete Co., Ltd., Wuhan 430074, China

**Keywords:** glass powder, glass aggregate, alkali-silica reaction (ASR), inhibitory effect

## Abstract

Detailed research is carried out to ascertain the inhibitory effect of waste glass powder (WGP) on alkali-silica reaction (ASR) expansion induced by waste glass aggregate in this paper. The alkali reactivity of waste glass aggregate is examined by two methods in accordance with the China Test Code SL352-2006. The potential of WGP to control the ASR expansion is determined in terms of mean diameter, specific surface area, content of WGP and curing temperature. Two mathematical models are developed to estimate the inhibitory efficiency of WGP. These studies show that there is ASR risk with an ASR expansion rate over 0.2% when the sand contains more than 30% glass aggregate. However, WGP can effectively control the ASR expansion and inhibit the expansion rate induced by the glass aggregate to be under 0.1%. The two mathematical models have good simulation results, which can be used to evaluate the inhibitory effect of WGP on ASR risk.

## 1. Introduction

Since the 1980s, the use of waste glass (WG) in concrete has been investigated in USA and Europe. With regard to concrete production, WG can be used as fine aggregate to replace sand after being crushed or as a supplementary cementitious material to replace cement after being ground. In the former case, glass exhibits double effects on properties of concrete, both positive and negative. If the replacement ratio is greater than 20%–30%, the mechanical properties will decrease as the replacement ratio increases [1]. However, the glass can effectively reduce the porosity of concrete [2,3] so as to improve the strength [4] and durability [5] if its replacement ratio is below 20%–30%. In the latter case, waste glass powder (WGP) can participate in pozzolanic reaction [6], which is beneficial to the properties of the binder. But the research on WG concrete is still at the preliminary stage [4] and needs further study.

WG contains a significant amount of alkali and silicon dioxide (reactive silica). It may lead to alkali-silica reaction (ASR) risk and concrete cracking, which is the main reason accounting for the early stagnation of research on the use of WG in concrete. Lots of research [1,2,3,6] indicates that WG may cause ASR expansion risk in concrete. However, most research has been conducted at the macro level, the micro aspects and the interface properties of the WG aggregate have drawn little attention, so is its inhibitory effect. Meanwhile, WGP can be used as a supplementary cementitious material, it has pozzolanic activity, and it can improve the strength of the cementitious system [7,8]. WGP seldom relates with ASR. The chemical reaction mechanism of WGP in concrete is still not well understood. The effect mechanism of WG aggregate and WGP on concrete still needs confirmation. Chemical reaction model and ASR risk of WGP under alkaline environment also need to be established. Only when the ASR mechanism of WG in concrete is fully understood, is it possible to take full advantage of WG and provide theoretic support for its wide application.

In this paper, ASR expansion and its mechanism of mortar containing WG as fine aggregate are investigated. The inhibitory effect of WGP as a replacement of cement on ASR is studied and verified by macro and micro tests. In addition, the results refer to used green glass, which is a soda-lime glass. Even slight changes in the compositions (white glass, brown glass, or others) could possibly lead to somewhat different results. The micro properties are also tested by scanning electron microscopy (SEM), which can be used to explain the ASR mechanism in depth.

## 2. Experimental

### 2.1. Raw Materials

Raw materials used in this paper include cement, glass, water and standard sand. The cement is 42.5 Portland cement, whose chemical compositions are listed in Table 1.

Soda-lime glass is employed in this work due to its popularity all over the world. WG aggregate is prepared by collected green glass after being cleaned, dried, crushed, and taken for a screening test according to GB/T14684-2001 Sand for construction [9]. The mass content of all sizes is listed in Table 2. The smaller particles (0.16–0.315 mm) occupy a certain proportion, *i.e.*, 15%.

**Table 1:** Chemical compositions of cement and glass (mass, %).

**Compositions**	SiO_2_	Al_2_O_3_	CaO	Fe_2_O_3_	MgO	BaO	F	SO_3_
**cement**	21.25	2.91	63.09	3.24	0.68	-	-	3.36
**glass**	68.41	2.58	9.52	0.39	0.39	0.50	0.30	0.13
**Compositions**	K_2_O	Na_2_O	TiO_2_	MnO	P_2_O_5_	Cr_2_O_3_	ZnO	Loss
**cement**	1.12	0.31	0.31	0.04	0.17	-	-	3.52
**glass**	2.14	14.10	0.11	0.31	0.07	0.12	0.89	0.04

**Table 2:** Gradation of glass aggregate.

**Sieve Diameter (mm)**	5–2.5	2.5–1.25	1.25–0.63	0.63–0.315	0.315–0.16
**Mass percentage (%)**	10	25	25	25	15

WGP refers to the glass particles with a maximum particle size below 300 μm in this work. WGP is prepared by grinding the WG fine aggregate in ball mill. Furthermore, WGP is divided into seven finenesses, named GA, GB, GC, GD, GE, GF and GG by controlling the grinding time. Its chemical compositions are also presented in Table 1. The main chemical compositions are 68.41% SiO_2_, 14.10% Na_2_O and 10.52% CaO, and it is a typical soda-lime glass (71% SiO_2_, 13%–15% Na_2_O and 10% CaO). WGP may have pozzolanic activity even cementing property due to high lime and silicon content. Meanwhile, it may also raise ASR risk of concrete due to the high alkali and silicon content.

**Figure 1:**
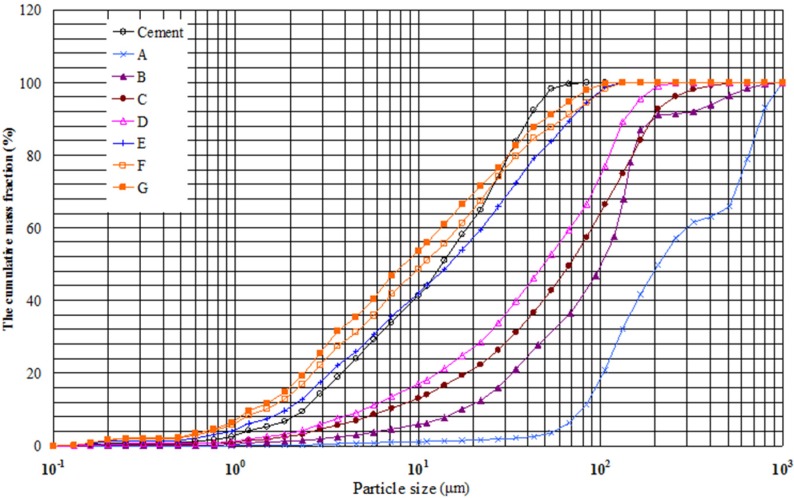
Particle size distribution of cement and the seven waste glass powders (WGPs).

Figure 1 shows the particle size distribution of the seven WGPs and cement. The seven WGPs become finer in turn. The gradation of GA and GB has larger leap and lacks particles with certain size, *i.e.*, 300–500 μm and 200–400 μm, respectively. The size distribution of GC is similar to that of GD. The particle size distribution of GE, GF and GG are similar to that of cement. No cement particle is larger than 100 μm, while the three WGPs contain small amounts of particles with a size higher than 100 μm. Furthermore, the specific surface area of the three WGPs is greater than that of cement because specific surface area depends on both particle size and particle shape of WGP. WGP contains more slender angular particles due to its fragile features, which leads to a higher specific surface area. Compared with cement, GF and GG have more particles below 20 μm and fewer particles with the size over 30 μm.

**Figure 2:**
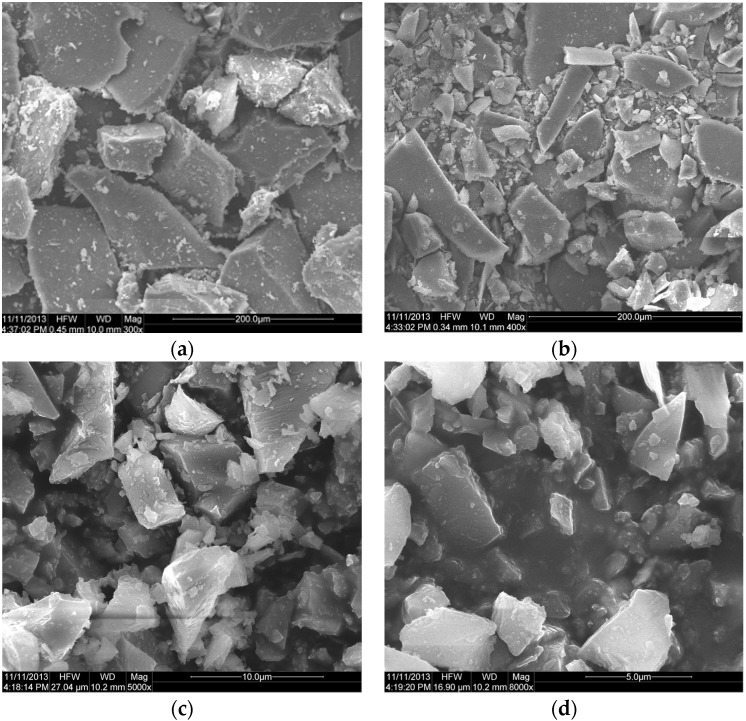
Particle morphology of WGPs: (**a**) GA, (**b**) GB, (**c**) GE and (**d**) GG.

The density of WGP is about 2.5 g/cm^3^ (Table 3), which is less than that of cement (about 3.1 g/cm^3^). The particle morphology of WGP is shown in Figure 2. The surface of WGP particles is relatively smooth, and its shape is irregularly angular. WGP particle size is consistent with the above analysis: the coarsest is GA, while the finest is GG.

**Table 3:** Physical characteristics of the seven waste glass powders (WGPs).

Samples	Average Particle Size (μm)	Specific Surface Area (m^2^/kg)	≥65 μm (%)	≥80 μm (%)	Density (g/cm^3^)
GA	209.2	31.00	94.33	89.97	2.48
GB	111.1	121.00	55.06	45.05	2.53
GC	68.29	217.00	51.58	44.53	2.49
GD	49.08	289.00	37.09	30.05	2.49
GE	14.94	658.00	11.55	6.63	2.49
GF	10.64	837.00	9.37	6.55	2.50
GG	8.20	903.00	5.92	2.75	2.48

**Figure 3:**
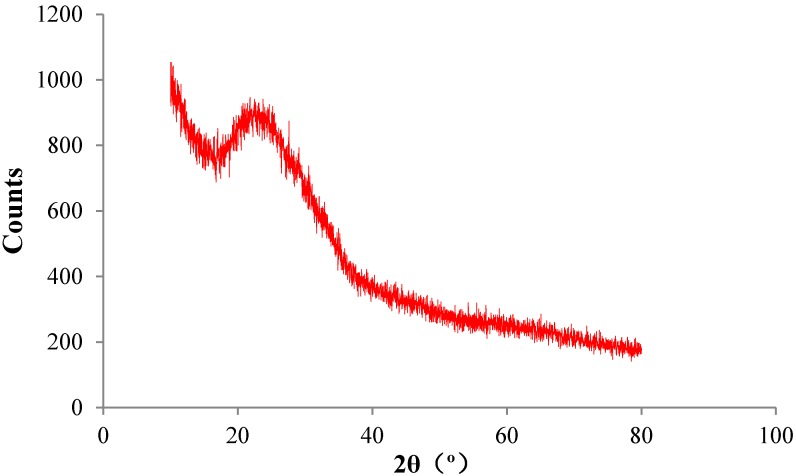
X-ray diffraction (XRD) pattern of WGP.

As can be seen from Figure 3, the X-ray diffraction (XRD) pattern of WGP is dispersive and exhibits a wide diffraction peak, named “bread peak”, near 25° different from the sharp peak of crystal, which indicates WGP is almost amorphous. WGP has great activity theoretically, and it is also related to its chemical construction, *i.e.*, the amorphous silica.

### 2.2. Experiments

In order to study the ASR activity and expansion development induced by WG aggregate, the mortar bar method (equivalent to ASTM C227 [10]) and the accelerated mortar bar method (equivalent to ASTM C1260 [11]) are used. The alkali content of cement is adjusted to be 0.6%. Mortars with water to cementitious materials ratio of 0.47 are prepared whose size is 25.4 × 25.4 × 285 mm^3^. The mix proportions are shown in Table 4. As for the accelerated mortar bar method, the specimens are demolded after 24 h and then placed in 1N NaOH solution at 80 °C to be totally immersed for 3, 7, 14 and 28 days. As for the mortar bar method, the specimens are demolded after 24 h and then stored in NaOH solution at 38 °C for 2 weeks, 1, 2, 3 and 6 months. According to the accelerated mortar bar method, the expansion rate less than 0.10% at 14 days is indicative of innocuous behavior, and the expansion rate larger than 0.20% is indicative of potentially deleterious expansion. According to the mortar bar method, the expansion rate less than 0.10% at 6 months is indicative of innocuous behavior. Tests are conducted according to regulations for aggregate alkali activity in test code SL352-2006 hydraulic concrete.

After ASR test, small clean specimens are taken out from the central of the mortars by a hammer and soaked in anhydrous ethanol to avoid carbonization before SEM observation, less than two weeks. The samples are dried for 2–3 h at 60 °C in a vacuum drying oven and placed in a vacuum coating machine for dehumidifying and gilding treatment.

**Table 4:** Mix proportions of mortar.

Samples	Cement (g)	Glass Powder (g)	Water (g)	Glass Aggregate (g)	Standard Sand (g)	NaOH (g)
*-WGP0	400	0	188	810	90	0.00
*-WGP10	360	40	188	810	90	0.00
*-WGP20	320	80	188	810	90	0.31
*-WGP30	280	120	188	810	90	0.85

Note: Number *, *i.e.*, A, B, C, D, E, F and G, on behalf of the incorporation of glass powder GA, GB, GC, GD, GE, GF, GG, the feature of glass powder GA–GG are corresponding to the glass powder in Table 3.

## 3. Results and Discussions

### 3.1. ASR of Glass Aggregate

The ASR risk of glass aggregate used to replace standard sand is tested by controlling the replacement ratio, *i.e.*, 0%, 10%, 30%, 50%, 70%, 90% and 100%, labeled as WG0, WG10, WG30, WG50, WG70, WG90 and WG100.

#### 3.1.1. The Accelerated Mortar Bar Method

**Figure 4:**
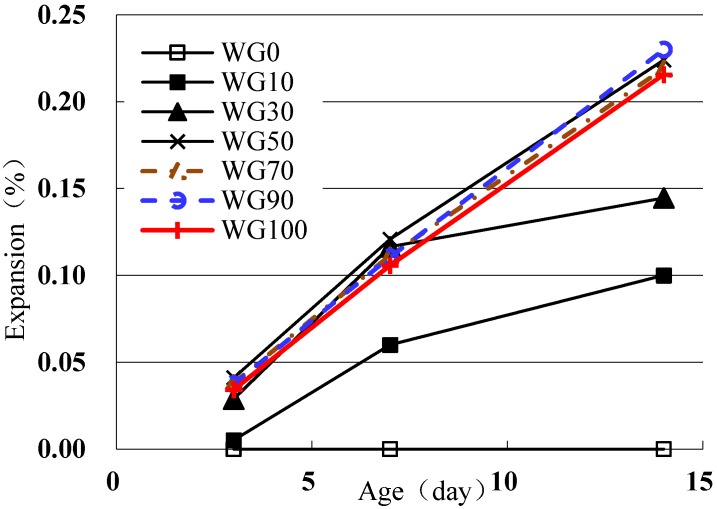
Alkali-silica reaction (ASR) expansion rate of the mortars with waste glass (WG) aggregate of different contents (accelerated mortar bar method).

According to Figure 4, WG aggregate belongs to alkali reactive aggregate. When glass aggregate content is higher than 30%, the expansion rate is larger than 0.10% at 14 days; when the content increases to 50%, the expansion rate of 14 days exceeds 0.20%; and, as the content increases further, the increasing trend of expansion rate obviously weakens and even decreases.

The expansion of 14 days reaches the maximum when WG content is 90%, which is the worst point for the ASR of concrete [12], but not 100%. When glass aggregate replacement ratio is below 90%, higher replacement ratio means more SiO_2_ and promotes more ASR gel. When the replacement ratio is higher than 90%, the increasing amount of SiO_2_ in the pore solution results in more OH^−^ absorption, which leads to insufficient OH^−^ to participate in ASR locally so that the amount of ASR gel decreases. WG aggregate has five size grades and contains partial WGP, which has inhibitory effect on ASR expansion because of its pozzolanic activity. Thus the expansion of samples containing more glass aggregate is a little lower. While some researchers found that ASR expansion shows increasing trend as glass aggregate replacement ratio increases [13]. This is probably due to the different test conditions, glass and cement types. Most researchers [13,14,15] have used a replacement ratio less than 50%, which is insufficiently detailed.

#### 3.1.2. The Mortar Bar Method

**Figure 5:**
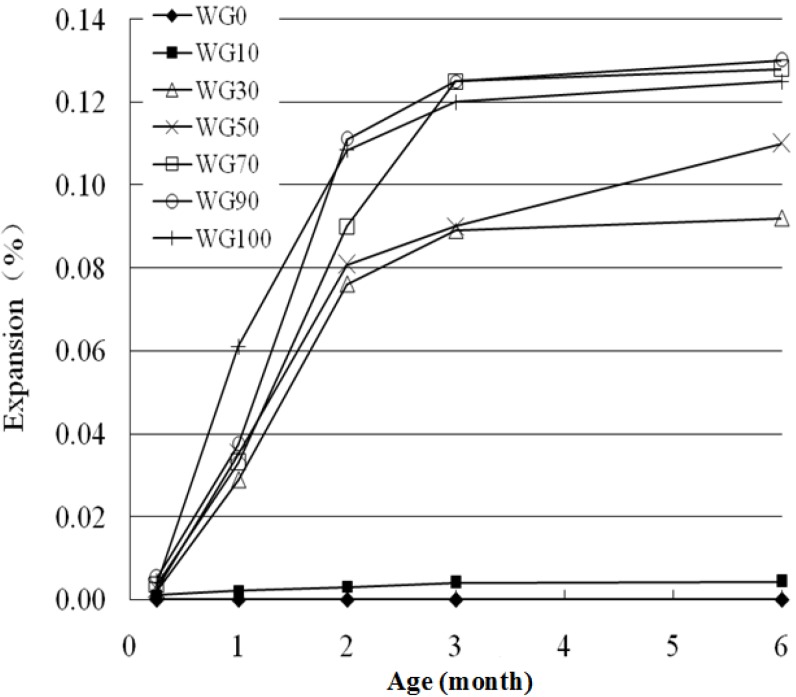
ASR expansion of the mortars with and without WG aggregate (mortar bar method).

The mortar bar method is also used and compared with the accelerated mortar bar method. The main difference is the curing temperature. In order to test the ASR activity rapidly, the period of the accelerated mortar bar method is 14 days, and the curing temperature is 80 °C. The temperature of the mortar bar method is 38 °C, which is closer to the actual engineering application [14], and is more reliable [15,16]. Figure 5 indicates that WG aggregate causes ASR expansion of the mortars.

As temperature can greatly improve the ASR, ASR expansion of the mortar bar method is much lower than that of the accelerated mortar bar method. When the replacement ratio of glass aggregate is greater than 30%, the expansion is larger than 0.05% at 90 days; when the replacement ratio increases to 50% or more, the expansion improves rapidly and reaches the most harmful level at 90% replacement ratio at 60, 90 and 180 days, which verifies the results observed by the accelerated mortar bar method.

### 3.2. The Inhibitory Effect of WGP on ASR

In order to analyze the inhibitory effect of WGP on ASR risk induced by WG aggregate, WGP is used to replace cement. The mix proportions are shown in Table 4.

#### 3.2.1. Effect of WGP Content

**Figure 6:**
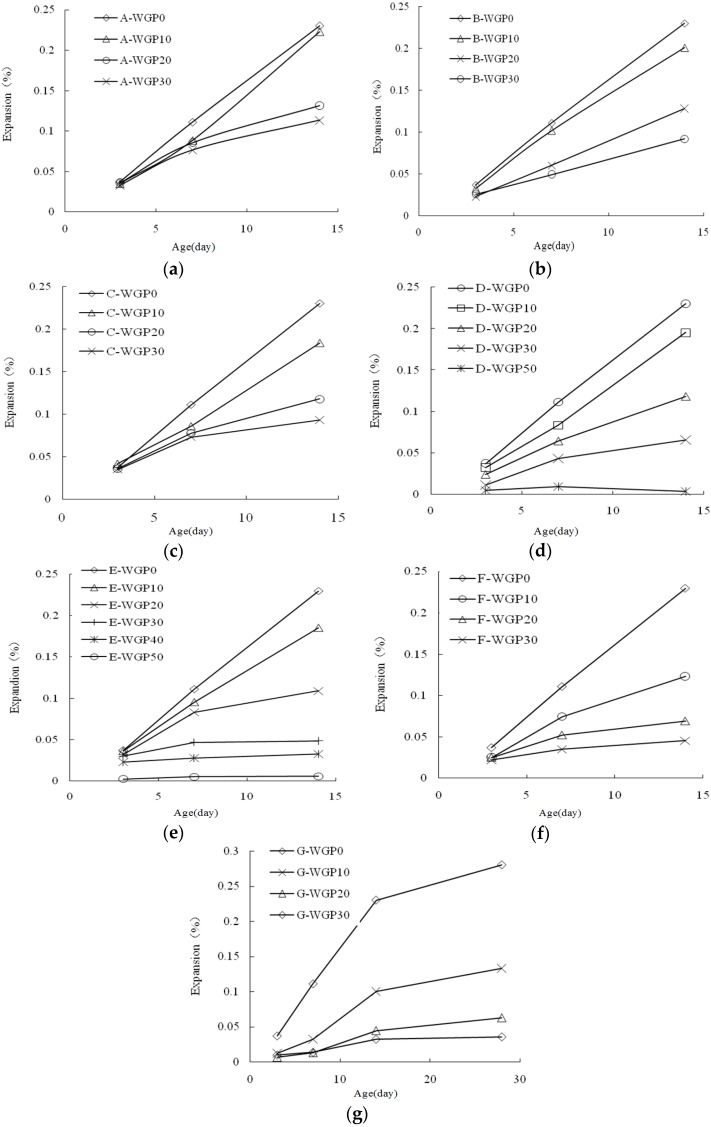
ASR expansion rate of the mortars containing different WGPs: (**a**) GA, (**b**) GB, (**c**) GC, (**d**) GD, (**e**) GE, (**f**) GF and (**g**) GG.

As shown in Figure 6, the expansion rate of all samples is reduced with the increase of WGP content. Pozzolanic reaction of WGP occurs firstly and Ca(OH)_2_ in solution is consumed, which leads to the reduction of ASR expansion. Meanwhile, calcium silicate hydrates (C–S–H) produced by WGP’s pozzolanic reaction combines with a part of alkali [17,18], which is useful to reduce ASR. The alkali of WGP released to pore solution is little, much lower than that of cement [14], which means the alkali released by the cementitious materials to pore solution reduces with the increase of WGP content. Different WGPs result in different inhibitory effect. For Group A, though the expansion rate is still reduced by WGP, the result is not significant. With respect to Groups B–E, when the content reaches 30%, the expansion can be kept in safe range. Groups F and G are able to achieve a high inhibitory effect when WGP content reaches 20%. The difference is related to the characteristics of WGP with different sizes.

The growth of ASR expansion rate is leveling off after mixing WGP, and the time-expansion curve converged gradually, especially for the samples containing 30% WGP. The pozzolanic activity of WGP is more obvious at later age, so it is more effective for reducing ASR.

#### 3.2.2. Effect of WGP Particle Size

The prevailing research shows that ASR expansion will decrease with the reduction of glass particle size when WGP is used as a cement substitute at the same replacement ratio. Recycled soda-lime glass aggregate shows the highest expansion in the size range of 1.18–2.36 mm when maintaining the same composition and mineralogy [19]. If the average particle size of WGP is less than 300 μm, there is no ASR expansion risk [19,20,21,22]. Some scholars have concluded that the blizzard ASR expansion does not occur as the average particle size is 0.6–1.18 mm or less, others argue this critical region is less than 0.15–0.30 mm [19]. Although there is still ASR expansion when WGP is extremely fine, the expansion rate is very low which has little influence on its use in concrete.

**Figure 7:**
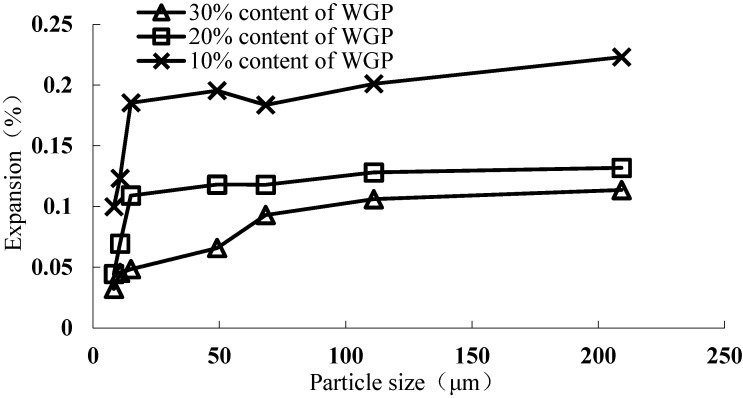
ASR expansion of the mortars containing WGPs with different particle size.

WGP with different particle size used to evaluate its inhibitory effect is shown in Figure 7. The finer the WGP, the higher is its inhibitory effect on ASR expansion [23]. If WGP size is less than 209.2 μm, it will not cause ASR damage and meanwhile has a certain inhibitory effect. For large WGP particles, SiO_2_ from the particle surface will be dissolved into the solution and react with Ca(OH)_2_ to form C–S–H gel until the local depletion. Thereafter, SiO_2_ react with alkali to generate ASR gel. However, as for fine WGP particles, WGP only conducts pozzolanic reaction and can be completely dissolved. Furthermore, WGP contains higher level of Ca^2+^, which is released for pozzolanic reaction and involved in the hydrated products [24]. So there may be not enough SiO_2_ to form ASR gel. And ASR gel swelling caused by WGP, which belongs to the uniform expansion, will not cause local destruction of concrete. The finer WGP has higher pozzolanic activity. The active aggregate is wrapped by WGP particles whose dissolution causes pozzolanic reaction to form C–S–H gel. Then, these small particles of C–S–H gel react with SiO_2_ of glass again to generate C–S–H gel with low Ca/Si ratio. ASR expansion rate can also be controlled in the safe level when the WGP content is 10%, 20% and 30% with its average particle size less than 8.2, 16.95 and 144.08 μm, respectively.

At the same time, it can be drawn from Figure 7 that when the WGP average particle size is less than 14.94 μm, the inhibitory effect becomes stronger with the decrease of the average particle size. Therefore WGP may have a critical region of inhibitory effect on ASR expansion. In this study, the critical average particle size is 14.94 μm (near 15 μm). However, as the fineness classification of WGP is not sufficient, it needs further verification.

**Figure 8:**
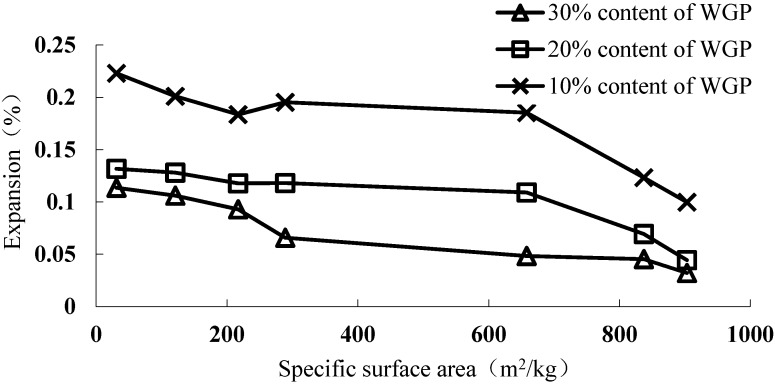
ASR expansion the mortars containing WGP with different specific surface area.

Figure 8 is the ASR expansion curves of mortars containing different WGPs with different specific surface area at 14 days. The specific surface area of WGP and the ASR expansion have an anti-correlation. When the material has larger surface area, the reaction area is larger and more atoms on the surface can participate in the reaction according to the atomic effective collision. The greater specific surface area of WGP results in more intense and faster pozzolanic reaction, and thus improves the inhibitory effect on ASR expansion of the system. In addition, the desirable effect can be achieved when WGP content is 10%, 20% and 30% with its specific surface area larger than 903, 698 and 97.61 m^2^/kg, respectively, according to the interpolation calculation.

#### 3.2.3. Effect of Temperature

**Figure 9:**
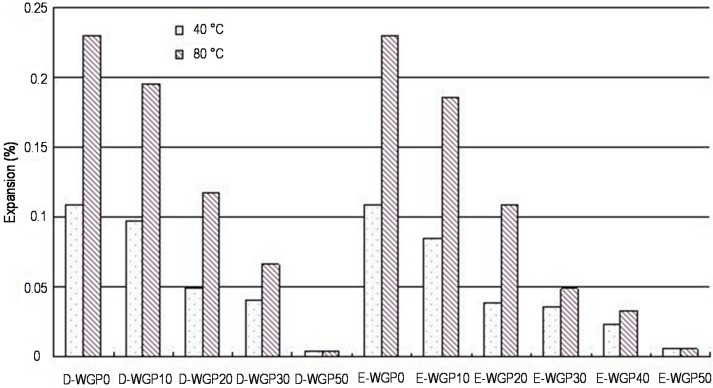
ASR expansion of different mortars under different temperatures at 14 days.

In practice, the actual temperature of concrete is different, so it is necessary to consider the effect of curing temperature on the ASR expansion. The ASR expansion under normal (40 °C) and high temperature condition (80 °C) is compared in Figure 9.

As high temperature can improve ASR, the development of ASR expansion under 40 °C is slower than that under 80 °C, and the expansion at 14 days is maintained at safe levels (<0.1%). The chemical reaction rate increases as well as ASR become more obvious along with the increase of curing temperature; meanwhile, higher temperature results in more alkali releasing to the pore solution and increasing ASR risk [25]. Nevertheless, some studies have shown that the high temperature cannot affect the final extent of ASR reaction.

As shown in Table 5, the temperature significantly affects the inhibitory effects of WGP on ASR expansion. As for low WGP content (<20%), the higher the temperature, the lower the inhibitory rate. As for high WGP content (30%), the result is opposite. Higher temperature also improves the pozzolanic reaction of WGP [26]. The pozzolanic reaction of WGP will reduce ASR, which are two mutually exclusive reactions. The interaction of them determines the ASR expansion rate of the system. When the WGP content is low, the promotion role of temperature on ASR is greater than pozzolanic reaction. However, it is the contrary way when WGP content is high. This is very similar to the role of fly ash. High temperature improves the solubility of fly ash, which promotes pozzolanic reaction, so it can control ASR effectively.

**Table 5:** The alkali-silica reaction (ASR) inhibition rate of glass powder at 14 days under different temperature (%).

**Temperature (**°C**)**	**D-WGP0**	**D-WGP10**	**D-WGP20**	**D-WGP30**	**D-WGP40**	**D-WGP50**
40	-	11.010	55.050	63.300	-	63.920
80	-	10.070	39.590	66.320	-	98.190
**Temperature (**°C**)**	**E-WGP0**	**E-WGP10**	**E-WGP20**	**E-WGP30**	**E-WGP40**	**E-WGP50**
40	-	22.020	64.220	66.970	78.900	95.410
80	-	19.420	52.610	78.960	85.820	97.610

#### 3.2.4. Inhibitory Efficiency of WGP

According to above analysis, there are two comprehensive factors (*i.e.*, average particle size and content of WGP) controlling the inhibitory effect on ASR expansion. Table 6 lists the inhibitory efficiency of different size and content of WGP in the entire system.

**Table 6:** The ASR expansion inhibition efficiency with different WGP contents (%).

Samples	WGP Content		
	10%	20%	30%
A-WGP	3.04	42.71	50.65
B-WGP	12.61	44.35	53.91
C-WGP	20.18	48.76	59.57
D-WGP	15.07	48.70	71.39
E-WGP	19.42	52.61	78.96
F-WGP	46.41	69.85	80.27
G-WGP	56.61	80.71	86.03

As can be seen from the above data characteristics, the reasonable law should be studied to provide a reference for the use of WGP. ASR expansion inhibitory rate *Y* of different WGP has logarithmic relationship with average particle size *D*, linearly related with WGP content *P*, so the model can be established as Equation (1):

(1) Y=lnln(A+BP+CD)

where, *Y* *is* the reducing rate of ASR expansion rate compared with the sample without WGP at 14 days, and *Y* = 1 − (ASR expansion of the sample with WGP**/**ASR expansion of the sample without WGP); *A*, *B* and *C* are Parameters; *P* is the WGP content; and *D* is the average particle size of WGP.

Using the software of Excel, parameters determined by fitting the data using linear regression, as calculated by Equation (2):

(2) Y=lnln(11.06182−27.1472P+0.019369D)

**Figure 10:**
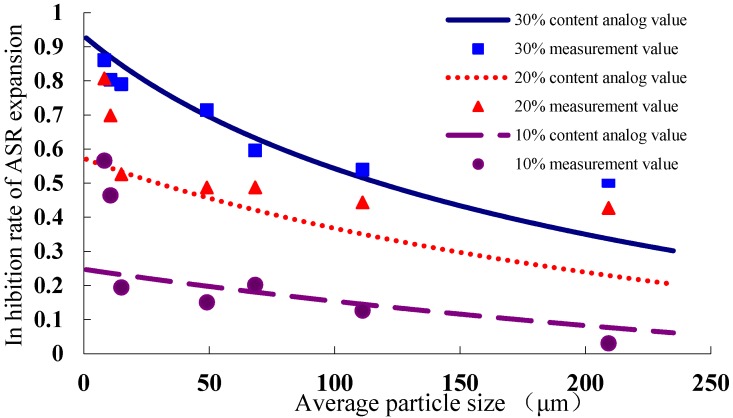
The inhibitory effect of different average particle size on ASR expansion.

As the fitting case is plotted (Figure 10), the inhibitory efficiency of WGP on ASR expansion has a certain relationship with WGP particle size and content. A rough estimate of ASR expansion can be obtained based on the size and content of WGP, which can provide the basis for the selection of WGP content and particle size. Basically, the smaller the particle size and the higher the WGP content, the higher the inhibitory effect. The simulation calculation shows that fine WGP can not solely control the ASR expansion into a safe range when WGP content is 10%; but ASR expansion rate can be controlled within safe level when WGP content is 20% and 30% with its average particle size less than 9.25 and 149.40 μm, respectively. The calculated results and interpolation calculations have some discrepancy, but the results are relatively more accurate and reliable than the interpolation calculation.

From the data characteristics above, there is a high correlation among the specific surface area and content of WGP, and inhibitory rate of ASR expansion, as Equation (3):

(3) Y=a+bP+cS

where *Y* is the reducing rate of ASR expansion rate compared with the sample without WGP at 14 days, *Y* = 1 − (ASR expansion of the sample with WGP**/**ASR expansion of the sample without WGP); *a*, *b* and *c* are Parameters; *P* is the WGP content; and *S* is the specific surface area of WGP.

Based on the linear regression fitting, the parameter can be obtained as Equation (4):

(4) Y=−0.12297+2.195983P+0.000412S

**Figure 11:**
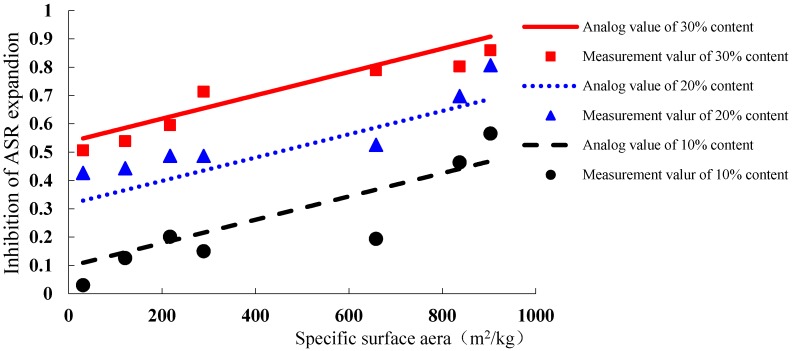
The inhibitory effect of different specific surface area on ASR expansion.

As shown in Figure 11, the inhibitory efficiency of WGP on ASR expansion has a linear correlation with the specific surface area and content of WGP. As surface area significantly affects WGP reactivity [27], the higher the surface area and the content of WGP, the better is the inhibitory efficiency. According to the simulation calculation, the ASR expansion can be controlled in a safe range when WGP content is 10%, 20% and 30% with its specific surface area larger than 1137.40, 604.37 and 71.34 m^2^/kg, respectively.

### 3.3. Microstructural Study

**Figure 12:**
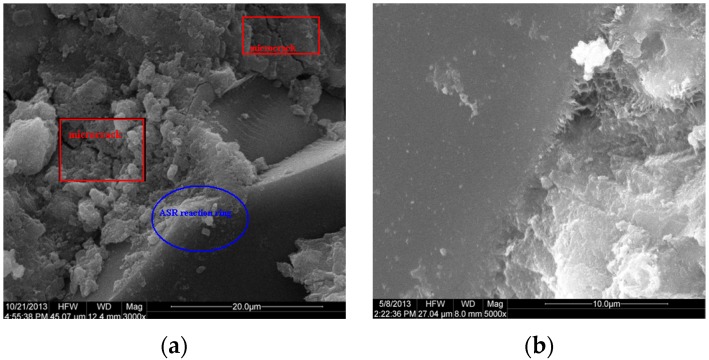
Scanning electron microscopy (SEM) images of mortars (**a**) without and (**b**) with 30% WGP.

Figure 12 is the SEM images of different samples. The ASR ring around the large glass aggregate particle and the microcrack could be found easily in Figure 12a, which shows the serious ASR expansion has taken place and caused cracking in the mortar. It is consistent with the results of the macro test. Compared with Figure 12a, there is no ASR ring or microcrack around the large glass aggregate, but it is webbed C–S–H in the sample containing 30% WGP (Figure 12b); the C–S–H gel may be generated by the pozzolanic reaction of WGP around the WG aggregate. The C–S–H may react with the SiO_2_ again to generate C–S–H with low Ca/Si ratio. ASR is a slow process that is later than the pozzolanic reaction, it generates C–S–H wrapping of the aggregate to improve the cementing property between the aggregate and the surroundings, and prevents ASR to some degree.

**Figure 13:**
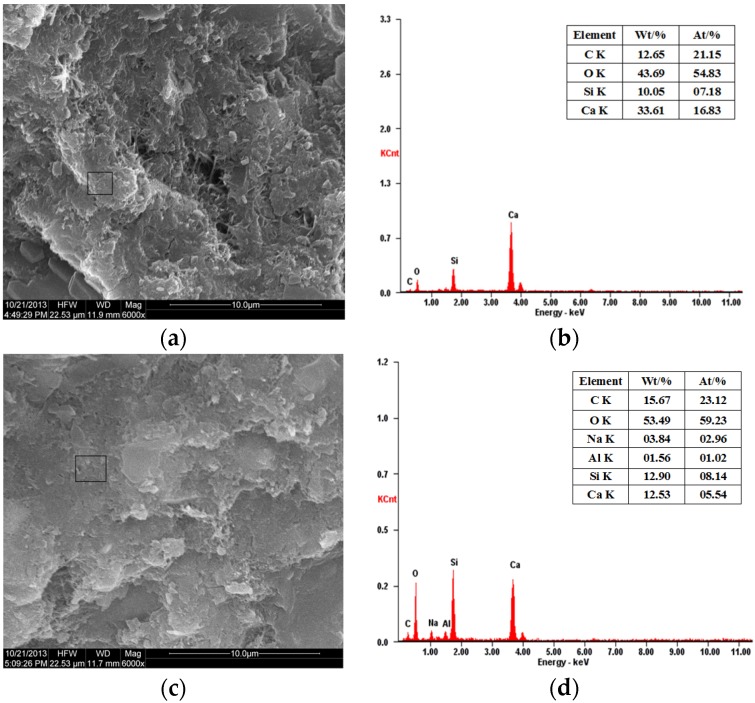
SEM and EDX results of mortars without and with 30% WGP: (**a**) SEM without WGP; (**b**) EDX without WGP; (**c**) SEM with WGP; and (**d**) EDX with WGP.

The Ca/Si ratio of C–S–H gel in the mortar with WGP is low and is close to 1 (Figure 13d). C–S–H gel with a low Ca/Si ratio is tobermorite whose gel strength is relatively high. It is the result of the pozzolanic reaction between reactive SiO_2_ of WGP and Ca(OH)_2_ of the initial cement hydration [28,29]. Additionally, excessive reactive SiO_2_ continues reacting with the C–S–H gel with high Ca/Si ratio to form C–S–H gel with low Ca/Si ratio. Besides, Figure 13d shows that the gel contains sodium, which is the chemical compositions of WGP. C–S–H gel with low Ca/Si ratio adsorbs the alkali metals strongly, which verifies that WGP preferentially reacts with the Ca(OH)_2_ to generate C–S–H gel and avoid ASR.

## 4. Conclusions

(1) WG aggregate has high alkali reactivity. The ASR expansion increases with the increase of the replacement ratio of glass aggregate, and reaches the maximum values at 90% replacement ratio.
(2) WGP can greatly reduce the ASR expansion induced by glass aggregate. The ASR expansion decreases with the increase of the content and fineness of WGP. When WGP size is less than 209.2 μm, it will add no ASR expansion and, meanwhile, it has a certain inhibitory effect on ASR expansion.
(3) The specific surface area of WGP and the ASR expansion have an anti-correlation, which leads the pozzolanic reaction more intense and faster and higher inhibitory effect on ASR expansion. The ASR expansion can be controlled in a safe range when WGP content is 10%, 20% and 30% with its specific surface area greater than 1137.40, 604.37 and 71.34 m^2^/kg, respectively, or with low average particle size according to the calculation.
(4) Higher temperature can promote both the pozzolanic reaction and ASR, and the inhibitory rate increases under high temperature for low WGP content (<20%), but decreases for high WGP content (30%).
(5) Microstructural study confirms the macro testing. There will be ASR reaction ring around the large glass aggregate particle and the microcrack in the mortar without WGP, but C–S–H gel with low Ca/Si ratio and no ASR will appear in the mortar with WGP.
